# Examining equity in the utilisation of psychiatric inpatient care among patients with severe mental illness (SMI) in Ontario, Canada

**DOI:** 10.1186/s12888-021-03419-4

**Published:** 2021-08-23

**Authors:** Claire de Oliveira, Joyce Mason, Rowena Jacobs

**Affiliations:** 1 Centre for Health Economics, University of York, Alcuin A Block, Heslington, York, YO10 5DD UK; 2 Hull York Medical School, University of York, Alcuin A Block, Heslington, York, YO10 5DD UK; 3 Institute for Mental Health Policy Research, Centre for Addiction and Mental Health, Toronto, Ontario M5S 2S1 Canada; 4 Institute for Health Policy, Management and Evaluation, University of Toronto, Toronto, Ontario M5T 3M6 Canada; 5 ICES, Toronto, Ontario M4N 3M5 Canada

**Keywords:** Severe mental illness, Equity, Inpatient care, Deprivation, Administrative health care data, Ontario

## Abstract

**Background:**

Severe mental illness (SMI) comprises a range of chronic and disabling conditions, such as schizophrenia, bipolar disorder and other psychoses. Despite affecting a small percentage of the population, these disorders are associated with poor outcomes, further compounded by disparities in access, utilisation, and quality of care. Previous research indicates there is pro-poor inequality in the utilisation of SMI-related psychiatric inpatient care in England (in other words, individuals in more deprived areas have higher utilisation of inpatient care than those in less deprived areas). Our objective was to determine whether there is pro-poor inequality in SMI-related psychiatric admissions in Ontario, and understand whether these inequalities have changed over time.

**Methods:**

We selected all adult psychiatric admissions from April 2006 to March 2011. We identified changes in socio-economic equity over time across deprivation groups and geographic units by modeling, through ordinary least squares, annual need-expected standardised utilisation as a function of material deprivation and other relevant variables. We also tested for changes in socio-economic equity of utilisation over years, where the number of SMI-related psychiatric admissions for each geographic unit was modeled using a negative binomial model.

**Results:**

We found pro-poor inequality in SMI-related psychiatric admissions in Ontario. For every one unit increase in deprivation, psychiatric admissions increased by about 8.1%. Pro-poor inequality was particularly present in very urban areas, where many patients with SMI reside, and very rural areas, where access to care is problematic. Our main findings did not change with our sensitivity analyses. Furthermore, this inequality did not change over time.

**Conclusions:**

Individuals with SMI living in more deprived areas of Ontario had higher psychiatric admissions than those living in less deprived areas. Moreover, our findings suggest this inequality has remained unchanged over time. Despite the debate around whether to make more or less use of inpatient versus other care, policy makers should seek to address suboptimal supply of primary, community or social care for SMI patients. This may potentially be achieved through the elimination of barriers to access psychiatrist care and the implementation of universal coverage of psychotherapy.

## Background

Severe mental illness (SMI) comprises a range of chronic and disabling conditions, such as schizophrenia, bipolar disorder and other psychoses. Although these disorders affect a small percentage of the population, they are associated with poor health, social and economic outcomes [1], including elevated risk of mortality [2–4], reduced life expectancy (13–30 years shortened life expectancy compared to the general population) [5], high costs of care and lost productivity [6–8], with psychiatric inpatient care accounting for a large portion of patients’ health care use. Poor outcomes are further compounded by disparities in access, utilisation, and quality of provision of care [9–12]. While there has been some debate between providing mental health treatment and care in hospitals versus community settings (primarily or even exclusively), there is no scientific evidence to suggest one type of care is superior to the other [13]. Instead, professional opinion and available studies support balanced care, which is essentially community-based, but where inpatient care can play an important supportive role [13]. This means that mental health services are provided in community settings close to the population served, and hospital stays are as brief as possible, arranged promptly, and employed only when necessary [13]. Despite this, little is known about the disparities in the utilisation of psychiatric inpatient care related to the socioeconomic status (SES) of patients with SMI.

Few studies have examined changes in the socio-economic equity of the utilisation of secondary care, where socio-economic equity is defined as equality of utilisation of secondary care between different deprivation groups after adjusting for need. Cookson et al., 2012 developed a robust method to measure changes in socio-economic equity of inpatient admissions and outpatient visits for specialist care from 2001 to 2008 using small-area level administrative data, which contained information on demand/need and supply variables [14]. The authors first estimated standardised utilisation ratios for various deprivation groups using ordinary least squares-based indirect standardisation for population, sex, age and disease prevalence. In particular, the authors regressed the number of inpatient admissions on population sex and age, disease prevalence, as well as demand/need and supply indicators for each year. The authors then estimated a pooled negative binomial model with a continuous measure of deprivation as the dependent variable to test for changes in the socio-economic equity of utilisation over time. They found there was no deterioration in the socio-economic equity in health care utilisation during the analysis period.

Employing the Cookson et al. (2012) methodology, White et al. (2014) examined the socio-economic equity in the utilisation of hospital care for patients with SMI using the Hospital Episode Statistics database from England [15]. Informed by a comprehensive literature review, the authors controlled for a series of relevant variables associated with SMI-related admissions. They found pro-poor socio-economic inequality in the utilisation of SMI-related psychiatric inpatient care (i.e., patients with SMI in more deprived areas were more likely to have psychiatric inpatient care than those in less deprived areas) and some evidence that this socio-economic inequality had decreased over time [15]. The authors posited that the pro-poor inequality might be due to the sub-optimal supply or quality of primary, community or social care for people with SMI in deprived areas, thus leading to higher use of inpatient care, rather than improved overall access to care in more deprived areas. In an effort to understand whether this pro-poor nature of psychiatric admissions was systemic or whether other factors such as differences in the funding and configuration of care played a role in their findings, they suggested that future research replicate their analysis using data from other jurisdictions with a similar health care system. This is an important issue to unravel as it affects policy recommendations that can be drawn in different countries.

Given the similarities between the English and Ontario health care systems, we sought to determine the type of inequality in psychiatric admission-related utilisation among patients with SMI in Ontario and whether these inequalities have changed over time. We hypothesise that there is pro-poor inequality in psychiatric admissions for patients with SMI in Ontario (i.e., patients with SMI in more deprived areas are more likely to be hospitalised than those in less deprived areas), in line with the findings from England. To undertake our analysis, we made use of the Cookson et al. framework, in line with White et al., and patient-level linked administrative health care data from Ontario, Canada’s most populous province. Similar to prior work, a major strength of this analysis is that it includes all psychiatric admissions for the adult population in Ontario, thus avoiding selection bias, which can occur in survey data.

## Methods

### Data

We used patient-level linked administrative health care data housed at ICES (formerly known as the Institute for Clinical Evaluative Sciences) in Toronto, Ontario, which includes data on most publicly funded health care services for all legal residents of Ontario. The ICES data repository includes the Ontario Mental Health Reporting System, which captures all psychiatric hospitalisations for individuals aged 16 and over that occur in designated mental health beds as well as the Discharge Abstract Database, which includes all non-adult psychiatric hospitalisations (i.e., for individuals under the age of 15) and adult psychiatric hospitalisations that occur in non-mental health designated beds. This data repository also includes the Registered Persons Database, a population-based registry of all legal Ontario residents; the Canada Census data, which includes neighbourhood-level data; the Immigration, Refugees and Citizenship Canada database, which includes information on all legal immigrants and refugees in Canada; the ICES Physician Database, which provides data (such as sex, age, place of practice, specialty, etc.) on all physicians practicing in Ontario; and the ICES Institution Database, which contains information on all health care institutions funded by the Ontario Ministry of Health.

### Patient cohort

We included all psychiatric admissions, from April 2006 to March 2011 (i.e., fiscal years 2006 to 2010), captured in the Ontario Mental Health Reporting System and the Discharge Abstract Database for individuals aged 15 and over with a *main* diagnosis of psychosis (Diagnostic and Statistical Manual of Mental Disorders 4th [DSM-IV] edition codes: 295.*, 297.1, 297.3, 298.8, 298.9; International Statistical Classification of Diseases and Related Health Problems 10th revision [ICD-10] codes: F20 (excluding F20.4), F22, F23, F24, F25, F28, F29, F53.1) or bipolar disorder (DSM-IV codes: 296* except 296.2, 296.3; ICD-10 codes: F30-F31) who were discharged before the end of the study period (March 31st 2011). (Although comorbid prevalence of substance use is high in individuals with SMI, psychiatric admissions with a main diagnosis of substance use-induced psychosis were not included in the analysis, as this type of psychosis is not typically long lasting.) For the purpose of this analysis, an inpatient psychiatric stay was defined as a completed continuous inpatient episode of care, which accounts for transfers between providers. We excluded all admissions among patients who were ineligible for public health insurance and/or not residing in the province during the analysis period and those who had missing data for variables of interest at the small area level of residence, such as neighbourhood-level deprivation, rurality of residence, and regional health authority of residence (these exclusions accounted for 3206 psychiatric admissions, i.e., 3.2% of 101,132 total admissions).

### Variables of interest

#### Dependent variable: psychiatric admissions

All relevant psychiatric admissions were aggregated at the dissemination area level, which in Canada corresponds roughly to the size of a neighbourhood and includes 400 to 700 persons [16]. We used the Statistics Canada’s Postal Code Conversion File to link admissions to dissemination areas in the Census data [17].

#### Main independent variable: deprivation

To obtain data on deprivation, we used information contained in the Ontario Marginalization Index, which explores multiple dimensions of marginalisation, such as residential instability, material deprivation, dependency, and ethnic concentration [18]. In turn, area-level deprivation was ascertained from the material deprivation score, which measures the inability of individuals and communities to access and attain basic material needs [18], and is made up of the following indicators: proportion of the population considered low-income; proportion of the population aged 15 and older who are unemployed; proportion of the population receiving government transfer payments; proportion of the population aged 20 and older without a high school diploma; proportion of households living in dwellings that are in need of major repair; and proportion of families who are lone parent families. This measure was available for 2006 and 2011. We used the 2006 value to derive the deprivation measure for 2006, 2007 and 2008, and the 2011 value to derive the deprivation measure for 2009 and 2010. The 2006 version was estimated using data from both the Canada Census short- and long-form questionnaires.^1^ In 2011, the federal government replaced the mandatory long-form census with the National Household Survey, which does not require mandatory reporting. The voluntary nature of this survey introduces the possibility of non-response bias among respondents. Therefore, the 2011 update does not use data from the National Household Survey but instead uses alternative data sources, such as Statistics Canada 2011 Canada Census Profiles data, the Registered Persons Database, the Immigration, Refugees and Citizenship Canada data, Statistics Canada Family Tax Return File and the Municipal Property Assessment Corporation data, to replace indicators formally based on the Census long-form questionnaire (this change in methodology appears to have had minimal impact on the construction of the score; nonetheless, caution should be applied when interpreting the results, namely when examining changes from 2008 to 2009).

#### Core, demand- and supply-side variables

We derived a series of variables for each dissemination area, which have been found to be risk factors for SMI-related hospital admissions and potential drivers of inequality [15]. To the extent possible, we tried to employ the same variables used by White and colleagues, which in turn were informed by their literature review (where this was not possible, we employed similar variables known to be associated with psychiatric admissions among individuals with SMI) [15]. We used the Registered Person Database to obtain data on core explanatory variables – total population aged 15 and older, and percentage of males and females by 5-year bands from 15 to 19 to 60–64 and wider age bands thereafter (65–74 and 75+). Demand-side (i.e., need) variables included SMI prevalence per 1000 individuals aged 15 and older (which was calculated using the hospitalisation databases), percentage of individuals identified as immigrants, and percentage of individuals identified as refugees (where these last two variables were determined through the Immigration, Refugee and Citizenship Canada database). Supply-side variables included an indicator of rural residency (where rural communities were defined as those with a population of 10,000 or less through the use of the Postal Code Conversion File), average minimum distance (in kilometres) to an acute care provider for individuals aged 15 and older, average minimum distance (in kilometres) to a mental health care provider for individuals aged 15 and older, general practitioner (GP) density per 1000 individuals aged 15 and older, and psychiatrist density per 1000 individuals 15 and older. The average minimum distance for all the provider variables was estimated using an “as the crow flies” distance method, which calculates great circle distances (in kilometres) from one place to another using latitude and longitude. We used each patient’s postal code and the postal code of the nearest hospital/provider within the regional health authority of residence, and estimated the shortest straight-line distance between the two. GP and psychiatrist densities by regional health authority were estimated using data in the ICES Physician Database.

Table 1 provides the descriptive statistics at the dissemination area level. Our sample included 97,926 admissions distributed across 96,834 dissemination areas. On average, about one SMI-related psychiatric admission occurred in each dissemination area over the study period.

**Table 1:** Descriptive statistics at the dissemination area level, April 2006 to March 2011

Number of dissemination areas = 96,834	Mean	SD
*Dependent variable*		
Admission Count	1.0	2.1
Admission Count (including admissions for major depression)	1.5	2.4
Admission Count (excluding admission for patients aged 75 and older)	1.0	2.0
*Deprivation variable*		
Material deprivation score	−0.1	0.9
*Core variables*		
Population aged 15 and older	557.1	418
% Age 15 to 19 - Males	8.6	3.4
% Age 20 to 24 - Males	8.1	3.0
% Age 25 to 29 - Males	7.6	3.0
% Age 30 to 34 - Males	7.7	3.3
% Age 35 to 39 - Males	8.7	3.4
% Age 40 to 44 - Males	9.9	3.3
% Age 45 to 49 - Males	10.4	2.9
% Age 50 to 54 - Males	9.2	2.7
% Age 55 to 59 - Males	7.9	2.6
% Age 60 to 64 - Males	6.4	2.6
% Age 65 to 74 - Males	8.7	4.1
% Age 75 and older - Males	6.7	5.0
% Age 15 to 19 - Females	7.8	3.1
% Age 20 to 24 - Females	7.5	3.1
% Age 25 to 29 - Females	7.3	3.4
% Age 30 to 34 - Females	7.6	3.5
% Age 35 to 39 - Females	8.5	3.4
% Age 40 to 44 - Females	9.6	3.3
% Age 45 to 49 - Females	10.1	3.0
% Age 50 to 54 - Females	9.0	2.8
% Age 55 to 59 - Females	7.8	2.8
% Age 60 to 64 - Females	6.4	2.8
% Age 65 to 74 - Females	9.0	4.2
% Age 75 and older - Females	9.1	7.2
*Need variables*		
SMI prevalence per 1000 per aged 15 and older	11.9	10.2
SMI prevalence per 1000 per aged 15 and older (with major depression)	16.6	12.4
% Refugee	1.9	3.0
% Immigrant	11.5	13.4
% Long-term resident	86.5	15.4
*Supply variables*		
% Rural residence	13.9	34.6
Average min. distance to any provider for aged 15 and older (km)	6.7	8.3
Average min. distance to acute provider for aged 15 and older (km)	6.9	8.3
Average min. distance to mental health provider for aged 15 and older (km)	13.5	23.7
GP density per 1000 per aged 15 and older	1.0	6.4
Psychiatrist density per 1000 per aged 15 and older	0.1	1.7

Legend: *SD* Standard deviation, *min.* minimum, *max.* maximum, *GP* General practitioner

Note: a higher value of the material deprivation score indicates higher level of deprivation

### Analysis

As this was a replication study, we followed White et al.’s approach [15] to model equity in the utilisation of psychiatric inpatient care, which in turn followed the methodology employed by Cookson et al. (2012) [14]. In line with Cookson et al. (2012), socio-economic equity in psychiatric inpatient care was defined as equality in the utilisation of psychiatric inpatient care between different small-area deprivation groups with the same need; the analysis was done in two stages.

In the first stage, we identified changes in equity over time across deprivation groups and geographic units of analysis using ordinary least squares-based indirect standardisation for need- and supply-side factors [19]. We estimated the following equation by ordinary least squares, separately for each year of data:

1 $$ {\mathrm{adm}}_i=\alpha +{D}_i\beta +{P}_i\varphi +{A}_i'\delta +{M}_i\ \lambda +{N}_i'\mu +{S}_i'\theta +{\varepsilon}_i $$

where i indexes the dissemination area of residence; adm denotes the number of SMI-related admissions; D represents the material deprivation score; P denotes a count of the population aged 15 and older; A denotes the vector of core explanatory variables (i.e., the percentage of the population in each age category by sex); M denotes SMI prevalence per 1000 individuals aged 15 and older; N denotes the vector of need variables; S denotes the vector of supply variables; and ε is an independent and identically distributed error term. The reference categories were total men aged 25–29, total women aged 25–29, percentage of individuals identified as long-term residents (i.e., not an immigrant or a refugee), and urban residency. In turn, need-expected number of psychiatric admissions in a given dissemination area and year were calculated as:

2 $$ \mathrm{a}{\hat{\mathrm{dm}}}_i=\kern0.28em \hat{\alpha}\kern0.28em +\overline{D}\hat{\varphi}\kern0.28em +{P}_i\hat{\beta}\kern0.28em +{A}_{i^{'}}\hat{\gamma}\kern0.28em +{M}_i\hat{\omega}\kern0.28em +\kern0.28em {N}_{i^{'}}\hat{\delta}\kern0.28em +\kern0.28em \overline{S}\hat{\iota \theta} $$

where the material deprivation and supply variables were fixed at the provincial-level mean values for that year to isolate (i.e., sterilise) the effect of material deprivation in the analysis and ensure the effects of higher supply were differentiated from those of higher need [18].

We examined material deprivation by quintile for each dissemination area. This differed from the White et al. study, which examined the percentage of the population that is income-deprived. Standardised utilisation ratios (SURs) were calculated for each deprivation quintile in each year by dividing the number of observed psychiatric admissions by the number of need-expected psychiatric admissions. A SUR of less than one suggests that utilisation in that deprivation quintile is lower than the utilisation that would be expected given the level of need. This may be due to poor access to psychiatric inpatient care (or good access to high quality primary, community or social care). Standardised utilisation rates were then calculated by multiplying the respective SUR by the provincial mean utilisation rate. All standardised utilisation rates were expressed per 100,000 individuals aged 15 and over. Standardised utilisation ratios and rates were also calculated at the Local Health Integration Network (LHIN) level, where LHINs were the regional health authorities in Ontario, which were responsible for planning, integrating and funding local health care. This was done by dividing the sum of observed psychiatric admissions in a given LHIN by the number of need-expected admissions in that LHIN.

In the second stage, we ascertained the nature of access to psychiatric inpatient care (i.e., pro-rich or pro-poor), and tested whether this had changed over time. To test for changes in equity of utilisation over each time period, the number of SMI-related psychiatric admissions for each geographic unit was modelled using a negative binomial model, controlling for core, demand- and supply-side variables. The second stage was used to help determine the direction of equity changes over time found in the first stage. In line with prior work [15], we estimated three sets of models: one with core explanatory variables only, one with core and demand explanatory variables and, finally, a full model, which included all variables (core, demand and supply).

#### Sensitivity analysis

We undertook three sensitivity analyses. First, we defined patients with SMI as those with a psychiatric admission for psychosis or bipolar disorder, which is in line with the definition of SMI in England. However, North American definitions of SMI tend to include psychosis, bipolar disorder *and* major depression. Thus, we re-did all analyses including patients hospitalised with major depression (DSM-IV codes: 296.2, 296.3; ICD-10 codes: F32, F33). Second, we replicated our analysis excluding all patients 75 years of age and older, as older patients may have received psychiatric care for dementia rather than SMI. Third, we re-estimated our model restricted to dissemination areas where patients had at least one admission to test the sensitivity of the results around ‘outlier’ dissemination areas, which contained a small number of individuals who had more than one admission. Given the change in the methodology used to derive the deprivation measure, we also replicated all analyses using the 2011 value only. The sensitivity analyses were done for all three sets of models.

## Results

### Changes in standardised utilisation ratios across deprivation quintiles

**Fig. 1:**
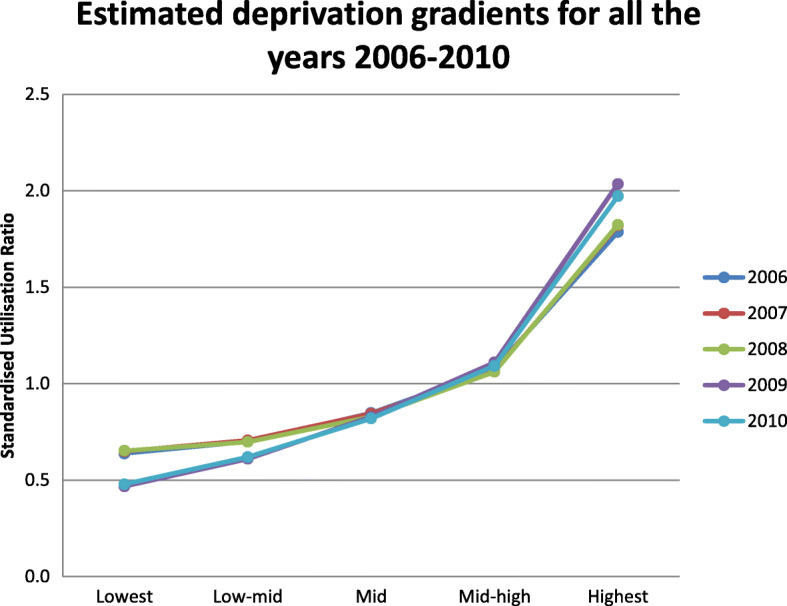
Standardised utilisation ratios by material deprivation score quintile from 2006 to 2010

**Fig. 2:**
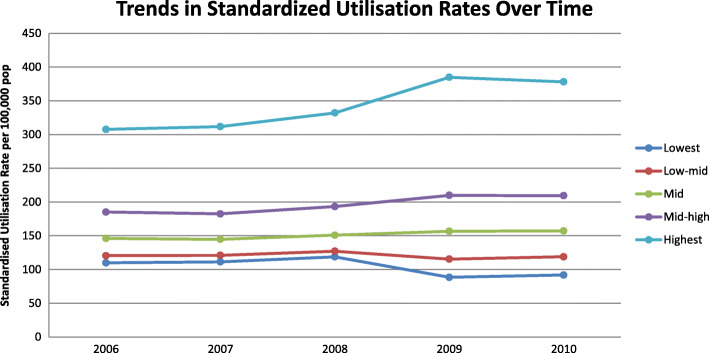
Standardised utilisation rates from 2006 to 2010 by material deprivation score quintile

Figure 1 provides the standardised utilisation ratios for each material deprivation quintile for each year. All lines are upward sloping, suggesting that equity of utilisation of psychiatric inpatient care is pro-poor, i.e., patients with SMI living in more deprived areas are more likely to be hospitalised than those living in less deprived areas. The highest deprivation groups have above-expected utilisation (SURs > 1), in line with findings in England. Changes over time occur mostly in the tails, i.e., in the lowest and highest material deprivation quintiles. In particular, the SURs for the less deprived group decrease over time, while the opposite holds for the most deprived group (though some caution should be applied in the interpretation of results). In other words, there is a worsening of conditions over time for the most deprived. Figure 2 provides trends over time in the SURs by material deprivation quintile. Trends are somewhat parallel between deprivation groups up until 2008, suggesting constant relative need for SMI care across groups. However, these trends change from 2008 onward, in particular for the highest deprived group, suggesting that need increases more rapidly for deprived patients compared to the other groups. This change is also likely due to the switch from the 2006 Census data to the 2011 Census data from 2008 to 2009. Full regression results are available upon request.

### Changes in standardised utilisation rates across LHINs over time

**Fig. 3:**
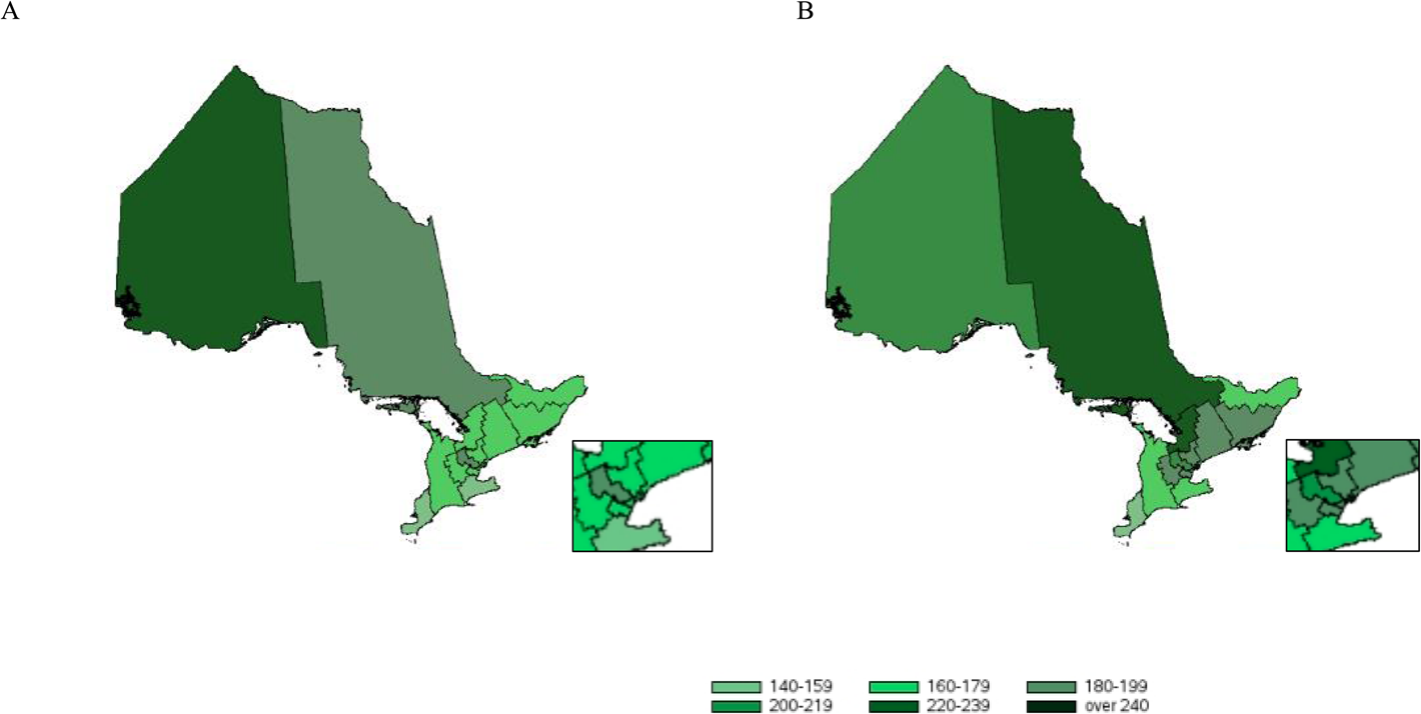
Standardised utilisation rates of SMI admissions per 100,000 individuals by Local Health Integration Networks in 2006 (**A**) and 2010 (**B**). Note: Local Health Integration Networks were the regional health authorities in Ontario. Both maps constitute original work and were produced by the authors using administrative health care data housed at ICES

Figure 3 depicts SURs per 100,000 individuals by LHINs for the first and last years of the analysis (i.e., 2006 and 2010). Overall, SURs are greater for areas with darker shades of green, such as very urban and very rural LHINs (i.e., the Toronto Central LHIN, which includes the city of Toronto, and the North West LHIN). From 2006 to 2010, SURs increase for urban and rural regions, namely LHINs in south central Ontario (i.e., the Toronto Central LHIN and Central LHIN), which includes many large cities, and northeastern Ontario (i.e., North Simcoe Muskoka LHIN and North East LHIN), which includes rural and remote areas of the province.

### Changes in equity over time

Table 2 includes the incidence rate ratios (IRRs) for the deprivation score estimated from the negative binomial regression models for all three sets of models and the sensitivity analyses. The IRR provides the proportional change in the number of psychiatric admissions associated with a one-unit increase in the independent variable. If the IRR is greater than one, then the independent variable will be positively associated with SMI admissions. For the main analysis, the IRR values associated with the deprivation score are all greater than one, which suggests that deprivation is positively associated with SMI admissions. For example, for the full model, if the deprivation score increases by one unit, the number of psychiatric admissions will increase by 8.1%. Unfortunately, given the differences in the deprivation measures, this value is not directly comparable to the one obtained in the White et al. study. In addition, the IRR values for the interaction term are just below one (and somewhat lower than the ones found in White et al.), indicating that the inequality of SMI admissions has become slightly less pro-poor over time; however, these values are not statistically significant. The inclusion of admissions for major depression does not change the IRR values by much. This is also the case when admissions for patients 75 years and older are excluded from the analysis, where the values do not change at all. However, when we model dissemination areas with at least one admission (roughly 43% of all disseminations areas), the values of the IRRs are substantially lower across all model configurations. Again, in all cases, all IRR values associated with the deprivation score are greater than 1 (and statistically significant in most cases); however, the values associated with the interaction term, while being greater than one, are no longer statistically significant, as found previously. When we replicate the analysis using the 2011 deprivation score only, we find our results are largely unchanged (results are available upon request).

**Table 2:** Summary of key values of the binomial regressions for the main analysis and sensitivity analyses

Model specification	Variable	Core model			Core + need model			Full model		
		IRR	SE	*P*-value	IRR	SE	*P*-value	IRR	SE	*P*-value
Main analysis	Material deprivation score	1.435	1.013	<0.0001	1.074	1.011	<0.0001	1.081	1.011	<0.0001
	Interaction term	0.987	1.014	0.367	0.994	1.012	0.649	0.993	1.012	0.559
Sensitivity analysis (including depression)	Material deprivation score	1.435	1.013	<0.0001	1.066	1.012	<0.0001	1.078	1.012	<0.0001
	Interaction term	0.987	1.014	0.367	0.992	1.013	0.502	0.989	1.012	0.380
Sensitivity analysis (excluding patients 75 and older)	Material deprivation score	1.435	1.013	<0.0001	1.074	1.011	<0.0001	1.081	1.011	<0.0001
	Interaction term	0.987	1.014	0.367	0.994	1.012	0.649	0.993	1.012	0.559
Sensitivity analysis (minimum 1 admission per dissemination area)	Material deprivation score	1.196	1.012	<0.0001	1.012	1.011	0.257	1.018	1.010	0.085
	Interaction term	1.000	1.012	0.974	1.012	1.010	0.222	1.010	1.010	0.277

Legend: *IRR* Incidence Rate Ratio, *SE* Standard error

Notes: “interaction term” denotes an interaction between the material deprivation score and a dummy variable for the year 2010; standard errors are clustered at the dissemination area level

## Discussion

We found above-expected utilisation in psychiatric admissions among the most deprived patients with SMI in Ontario, suggesting pro-poor inequality in the utilisation of psychiatric inpatient care. In other words, patients with SMI in more deprived areas were more likely to be hospitalised. This was particularly the case for very urban areas, where many patients with SMI reside, and very rural areas, where access to mental health-related health care is problematic. This finding could be, in part, due to barriers to access care as well as the suboptimal supply of primary, community or social care in those areas. After controlling for relevant variables through regression modelling, we found that this inequality remained unchanged over time.

Few studies have examined equity in the utilisation of secondary care, in particular psychiatric inpatient care. One study from England examined changes in socio-economic equity of inpatient admissions and outpatient visits for specialist care over an eight-year period and found no change in socio-economic equity in health care utilisation [14]. Specifically focusing on mental health-related care, and using a similar approach to the previous study, White et al. (2014) examined socio-economic equity in the use of inpatient psychiatric care for patients with SMI in England from 2006 to 2010 [15]. The authors found pro-poor inequality in the utilisation of psychiatric admissions, likely a result of suboptimal supply of primary, community or social care, and an improvement over time, albeit small. However, based on their results, the authors could not conclude whether the pro-poor nature of psychiatric admissions for SMI patients in England was systemic or whether other factors (such as funding differences and/or configuration of care) explained their findings.

We replicated the White et al. analysis and found that the inequality in access to psychiatric inpatient care (i.e, admissions) in Ontario was also pro-poor. In particular, we found that a one unit increase in the deprivation score increased the number of psychiatric admissions by about 8.1%, even after controlling for relevant covariates. We suspect our findings may also be systemic. On one hand, the funding structure in both jurisdictions is similar. Both Ontario and England have publicly funded health care systems, which provide universal coverage for hospital-based care. On the other hand, the configuration of care is somewhat different in both jurisdictions. For example, psychotherapy is not currently covered under the public health insurance plan in Ontario, while it is in England. However, this likely does not contribute much to any differences we may observe since psychotherapy is not the mainstay of care for patients with SMI. Moreover, while there is more reliance on psychiatrist-provided care in Ontario compared to England, there are significant barriers to accessing these specialists [20, 21]. Thus, it is not clear whether/how our results may have been affected by these differences. It will be important to re-examine this issue once psychotherapy coverage is in place in Ontario and more recent data are available. Furthermore, it is important to understand whether geographic drift may occur over time where individuals with SMI move to more deprived populated areas. Previous research using population-based, longitudinal administrative health care data from Manitoba, Canada, suggests that the odds of moving are higher for individuals with SMI compared to those with no mental illness, although there were no statistically significant differences in rural-to-rural or rural-to-urban migration [22].

While there has been some debate regarding the optimal use of inpatient versus primary, community and social care to treat individuals with SMI, improving access to these types of care may help address these inequalities. Thus, policy makers should seek to implement strategies that address the suboptimal supply of primary, community or social care for SMI patients. Our results suggest that more attention is required for patients with SMI living in areas where there are large inequalities, such as those in very urban and rural areas. Although not examined in this paper, this could potentially be achieved by eliminating barriers to access psychiatrist care, for example, by providing incentives to psychiatrists to take on more complex cases, such as patients with SMI. Previous work in Ontario has shown that psychiatrists in high supply urban areas tend to see healthier patients [20]. In addition, other measures may include encouraging further the use of telepsychiatry in more rural/remote areas, as prior work has evidenced low use of these services (compared to need) in Northern Ontario [23], as well as implementing universal coverage of psychotherapy. There may also be a need to address the social determinants of health for individuals with SMI by tackling issues such as housing and homelessness.

Similar to prior work, a major strength of this analysis is that it included all psychiatric admissions for adults with SMI residing in Ontario, thus avoiding selection bias that can be present in survey data. Given Ontario’s one-tier health care system (for publicly insured services, such as hospital-based care), there are likely no patients who obtain inpatient care privately. In addition, and to align with North American definitions of SMI, we examined all patients with an admission for psychosis, bipolar disorder and major depression, thus extending previous work.

Nonetheless, our work is not without limitations. Given the use of area-level data, the ecological nature of the data represents the main limitation, as these data cannot account for all socioeconomic variation at the individual level. Other limitations pertain to the data. We were not able to account for all relevant variables associated with SMI-related psychiatric hospitalisations. We were not able to extend our analysis beyond 2011, as deprivation data were not available for more recent years at the time of the analysis. Furthermore, the deprivation score for 2011 was estimated using alternative available data sources and thus not directly compared to the 2006 measure. We could not account for patients who did not access the health care system and were not captured in the hospitalisation databases (for example, homeless individuals). We used an “as the crow flies” method to estimate the distance between each patient and the nearest provider within the regional health authority of residence. While this method was easier to apply, it may not reflect actual distances that patients travel to access care, as roads were not considered. Furthermore, there may have been cases where individuals accessed a health care provider in a regional health authority that was not their regional health authority of residence due to greater proximity, and thus were assigned a longer minimum average distance than what may have occurred in reality. Future research should seek to address these limitations. Moreover, given that this analysis only used data from one Canadian province (i.e., Ontario), future research should seek to extend it to all of Canada. Finally, we did not account for spatial autocorrelation, which would have enabled us to understand whether psychiatric admissions in a given area were correlated with those in neighbouring areas [24, 25]. This should be the focus of future work on this topic.

## Conclusions

In sum, we found pro-poor inequality in the utilisation of psychiatric admissions among patients with SMI in Ontario; in other words, patients with SMI in more deprived areas were more likely to be hospitalised than those less deprived areas. This was particularly the case among more urban areas, where many patients with SMI reside, as well as more rural/remote areas, where access to mental health-related care is problematic. Moreover, our results suggest these inequalities have not changed over time. Although there has been some debate regarding the use of inpatient versus primary, community and social care to treat individuals with SMI, improving access to these types of care may help address these inequalities. Thus, policy makers should seek to address suboptimal supply of primary, community or social care for SMI patients. Some potential ways to achieve this may include the elimination of barriers in the access to psychiatrist care, for example, by encouraging psychiatrists to take on more complex cases, such as patients with SMI, the increase in the use of telepsychiatry in rural/remote areas, and the implementation of universal coverage of psychotherapy.

## Data Availability

The data that support the findings of this study are available from ICES but restrictions apply to the availability of these data, which were used under license for the current study, and so are not publicly available.

## Abbreviations

SMI: Severe mental illness
SES: Socioeconomic status
GP: General practitioner
SUR: Standardised utilisation ratio
LHIN: Local Health Integration Network
IRR: Incidence rate ratio

## Footnotes

1 The Census short-form questionnaire includes information on respondents’ sex, age, marital status and mother tongue. The Census long-form questionnaire, asked of a random sample of 1 in 4 private dwellings, includes sociocultural information, mobility, parents’ place of birth, education, labour market activities and housing, in addition to the information included in the short-form questionnaire. For more information, see: http://www23.statcan.gc.ca/imdb/p2SV.pl?Function=getSurvey&SDDS=3901
